# Characterization of the periplasmic redox network that sustains the versatile anaerobic metabolism of *Shewanella oneidensis* MR-1

**DOI:** 10.3389/fmicb.2015.00665

**Published:** 2015-06-29

**Authors:** Mónica N. Alves, Sónia E. Neto, Alexandra S. Alves, Bruno M. Fonseca, Afonso Carrêlo, Isabel Pacheco, Catarina M. Paquete, Cláudio M. Soares, Ricardo O. Louro

**Affiliations:** Inorganic Biochemistry and NMR Laboratory, Instituto de Tecnologia Química e Biológica António Xavier, Universidade Nova de LisboaOeiras, Portugal

**Keywords:** dissociation constant, electron transfer, electrostatics, extracellular respiration, paramagnetic NMR, periplasmic cytochromes, *Shewanella oneidensis* MR-1

## Abstract

The versatile anaerobic metabolism of the Gram-negative bacterium *Shewanella oneidensis* MR-1 (SOMR-1) relies on a multitude of redox proteins found in its periplasm. Most are multiheme cytochromes that carry electrons to terminal reductases of insoluble electron acceptors located at the cell surface, or *bona fide* terminal reductases of soluble electron acceptors. In this study, the interaction network of several multiheme cytochromes was explored by a combination of NMR spectroscopy, activity assays followed by UV-visible spectroscopy and comparison of surface electrostatic potentials. From these data the small tetraheme cytochrome (STC) emerges as the main periplasmic redox shuttle in SOMR-1. It accepts electrons from CymA and distributes them to a number of terminal oxidoreductases involved in the respiration of various compounds. STC is also involved in the electron transfer pathway to reduce nitrite by interaction with the octaheme tetrathionate reductase (OTR), but not with cytochrome *c* nitrite reductase (ccNiR). In the main pathway leading the metal respiration STC pairs with flavocytochrome *c* (FccA), the other major periplasmic cytochrome, which provides redundancy in this important pathway. The data reveals that the two proteins compete for the binding site at the surface of MtrA, the decaheme cytochrome inserted on the periplasmic side of the MtrCAB–OmcA outer-membrane complex. However, this is not observed for the MtrA homologues. Indeed, neither STC nor FccA interact with MtrD, the best replacement for MtrA, and only STC is able to interact with the decaheme cytochrome DmsE of the outer-membrane complex DmsEFABGH. Overall, these results shown that STC plays a central role in the anaerobic respiratory metabolism of SOMR-1. Nonetheless, the trans-periplasmic electron transfer chain is functionally resilient as a consequence of redundancies that arise from the presence of alternative pathways that bypass/compete with STC.

## Introduction

*Shewanella oneidensis* MR-1 is a Gram-negative bacterium that can use a wide range of terminal electron acceptors in the absence of oxygen, including fumarate, nitrite, nitrate, trimethylamine oxide (TMAO), dimethyl sulfoxide (DMSO), sulfur compounds and a variety of metal compounds including radionuclides (Myers and Nealson, 1988; Nealson and Saffarini, 1994; Myers and Myers, 2000; Gralnick and Newman, 2007; Burns and DiChristina, 2009). This metabolic versatility has made SOMR-1 a target of biotechnological research for the development of novel bioremediation processes and generation of electricity in MFC (Lovley, 2006; Logan and Rabaey, 2012). Electron transfer from SOMR-1 to extracellular substrates relies on the conduction of electrons from the cytoplasm to the cell surface via a periplasmic network of redox proteins dominated by *c*-type cytochromes (Heidelberg et al., 2002; Hau and Gralnick, 2007). With the exception of thiosulfate and TMAO reduction, all forms of anaerobic respiration described in SOMR-1 are routed via the tetraheme *c*-type cytochrome CymA, anchored to the inner membrane of the cell (Myers and Myers, 1997; Saffarini et al., 2002). This protein collects electrons from the menaquinone pool in the cytoplasmic membrane and distributes them among periplasmic proteins. These proteins can be either terminal reductases or periplasmic redox shuttles that transfer electrons to outer-membrane reductases (Myers and Myers, 1997; Saffarini et al., 2002; Schwalb et al., 2003). Nevertheless, the detailed organization of the trans-periplasmic redox network remains to be completely elucidated (Hartshorne et al., 2009). In anaerobic conditions, the most abundant periplasmic *c*-type cytochromes in SOMR-1 are the STC and the FccA (Tsapin et al., 2001). It was recently shown that both STC and FccA can accept electrons from CymA and transfer them to outer-membrane metal reductases, which lead to the identification of two independent redox pathways across the periplasm (Fonseca et al., 2013). Although this finding confirmed the functional redundancy already observed for other multiheme cytochromes (Coursolle and Gralnick, 2010; Richardson et al., 2012), the physiological reason for this is still unknown. One of the most explored and well described anaerobic respiratory pathways in SOMR-1 is Fe(III) reduction, which involves the outer membrane MtrCAB–OmcA complex (Myers and Nealson, 1988; Myers and Myers, 1997, 2000; Pitts et al., 2003). However, less is known about the periplasmic electron transfer network that delivers electrons to the homologous complexes, MtrDEF and DmsEFABGH. While the DmsEFABGH complex is responsible for the extracellular respiration of DMSO, the specific physiological function of MtrDEF is still unclear. It appears to play a similar role to MtrCAB–OmcA complex because of their high similarity (Fredrickson et al., 2008; Coursolle and Gralnick, 2010).

Another important respiratory process carried out by SOMR-1 is the conversion of nitrogen compounds to ammonia (Myers and Nealson, 1988; Nealson and Saffarini, 1994). This respiratory pathway involves the OTR and the cytochrome *c* nitrite reductase (ccNiR). OTR is described as an efficient nitrite and hydroxylamine reductase and ccNiR catalyzes the reduction of nitrite (Einsle et al., 2002; Atkinson et al., 2007). It was previously demonstrated that CymA is essential for nitrite reduction (Schwalb et al., 2003), but nothing is known about the routes of electron transfer to OTR and ccNiR. In order to determine if STC and FccA mediate the electron transfer from CymA to the outer-membrane complexes MtrDEF and DmsEFABGH, protein interactions studied by Nuclear magnetic resonance (NMR) and UV-visible spectroscopy were performed. NMR spectroscopy together with protein electrostatic surface potential calculations was also used to explore the ability of STC and FccA transfer electrons to OTR and ccNiR (Mowat et al., 2004; Youngblut et al., 2012). These results showed that the functional redundancy between STC and FccA appears to be restricted to the interaction with the MtrCAB–OmcA complex. Furthermore, the data showed that STC plays the major role in connecting CymA with the DmsEFABGH complex and OTR, but is not involved in interactions neither with ccNiR nor with MtrD.

## Materials and Methods

### Cloning and Expression of the Genes of Interest

The vectors containing *dmsE* and *mtrD* genes were kindly provided by Dr. Liang Shi from the Pacific Northwest National Laboratory (Richland, WA, U.S.A.). A stop codon was inserted at the 3′ end of each gene, allowing the removal of the V5 epitope and the 6xHis-tag sequence at the C-terminus of the proteins. The lack of these sequences eliminated concerns about proper folding of the protein in the presence of the 6xHis-tag at the C-terminus. The cloning vector pHSG298 (Takara Bio), containing the *ccnir* gene with the wild-type N-terminal signal peptide replaced by the signal peptide from the SOMR-1 protein STC, was gently provided by Dr. Sean J. Elliott from Boston University. The *otr* gene was amplified from genomic DNA of SOMR-1 and cloned into the pBAD202/D-TOPO vector following the instructions from the supplier (pBAD202 directional TOPO Expression Invitrogen KIT) and according to the work of Shi et al. (2005). The primers used are reported in **Table 1.**

**Table 1:** Primers used in this study.

Primer	Sequence (5′-3′)
DmsE_Stop_Forw	GCGGCAGCAATTTTGCCCGTTGAGGCGAGCTCAAGCTTGAAGGTAAGCC
DmsE_Stop_Rev	GGCTTACCTTCAAGCTTGAGCTCGCCTCAACGGGCAAAATTGCTGCCGC
MtrD_Stop_Forw	AAGTTGCTGCAGAGATAAGGCGAGCTCAAGCTTGA
MtrD_Stop_Rev	TCAAGCTTGAGCTCGCCTTATCTCTGCAGCAACTT
OTR_Stop_Forw	CACCTAAGAAGGAGATATACATCCCATGAA
OTR_Stop_Rev	TTATTGCTTATGTTTAGGGCCTTGTTTGTT


### Bacterial Strains and Growth Conditions

*Shewanella oneidensis* JG207 strain (knockout strain of the *fccA* gene), kindly provided by Prof. Dr. Johannes Gescher from Karlsruher Institut für Technologie (Karlsruher, Germany), was transformed with the vector containing the *otr* gene. The vectors containing the *dmsE* and *mtrD* genes were separately transformed in SOMR-1. *S. oneidensis* cells were grown at 30°C in Terrific Broth (TB) containing 50 μg/ml kanamycin in 5 l Erlenmeyer flasks containing 2 l of medium and 1:100 inoculum volume, at 130 rev./min. Protein expression was induced by addition of L-arabinose: 1 mM (for the strains over-expressing DmsE and OTR) and 2 mM (for the strain over-expressing MtrD) after 6–8 h of growth. After induction, cells continued to grow for 16 h, until harvesting. Bacterial cells were harvested by centrifugation at 11,325 *g* for 10 min, at 4°C. In this study the strain SOMR-1 was used to express ccNiR employing the growth conditions previously reported in the literature (Judd et al., 2012). The vectors, strains and growth conditions to express MtrA, STC, FccA, and CymA were as previously described (Fonseca et al., 2009, 2013).

### Protein Purification

The cell pellets were resuspended in 20 mM Tris-HCl (pH 7.6) containing protease inhibitor cocktail (Roche) and DNase I (Sigma). The disruption of the cells was achieved by a passage through a French Press at 1000 psi. Membranes and cell debris were removed by centrifugation at 219,000 *g* for 1 h, at 4°C, and the supernatant containing the soluble protein fraction was dialyzed overnight against 4 l of 20 mM Tris-HCl (pH 7.6). These fractions were concentrated in ultrafiltration cells, using a 10 kDa cut-off membrane. The fractions containing each of the target proteins were loaded onto a diethylaminoethyl (DEAE) column (GE Healthcare) pre-equilibrated with 20 mM Tris-HCl (pH 7.6). A gradient from 0 to 1 M NaCl was applied. The fractions containing MtrD were eluted at 150 mM NaCl, while DmsE and OTR were eluted at 200 mM NaCl. The fractions containing DmsE and MtrD were concentrated and loaded onto a HTP (hydroxyapatite) column (Bio-Rad Laboratories) pre-equilibrated with 10 mM potassium phosphate buffer (pH 7.6) and gradient from 10 mM to 1 M. DmsE and MtrD were eluted at 100 and 150 mM of potassium phosphate buffer (pH 7.6), respectively. The final purification step used a Superdex 75 column (GE Healthcare) pre-equilibrated with 20 mM potassium phosphate buffer (pH 7.6) and 100 mM NaCl. The fraction resulting from the DEAE column containing OTR was concentrated and loaded onto a Q-Sepharose column (GE Healthcare) previously equilibrated with 20 mM Tris-HCl (pH 7.6). A salt gradient from 0 to 1 M NaCl was applied and this protein was eluted at 150 mM NaCl. The fraction containing OTR was concentrated and loaded onto a HTP column pre-equilibrated with 10 mM potassium phosphate buffer (pH 7.6). A gradient from 10 mM to 1 M of potassium phosphate buffer (pH 7.6) was applied, and pure OTR was eluted at 100 mM. The purification of STC, FccA, CymA, and ccNiR cytochromes was performed as described in the literature (Judd et al., 2012; Fonseca et al., 2013). The recombinant TEV protease was removed from the fraction containing ccNiR using a Superdex 75 column pre-equilibrated with 20 mM potassium phosphate buffer (pH 7.6) with 150 mM KCl. All the chromatographic fractions were analyzed by SDS/PAGE stained for heme proteins (Francis and Becker, 1984) and by UV-visible spectroscopy to select those containing the target proteins. Proteins were considered pure when having an absorbance ratio Soret Peak/A280nm higher than 3.5, and when showing a single band in Coomassie staining SDS/PAGE gels. The identity of DmsE, MtrD, and OTR was confirmed by mass spectrometry and N-terminal sequencing.

### NMR Sample Preparation and Titrations

Stock samples of DmsE, MtrD, OTR, ccNiR, STC, and FccA in 20 mM potassium phosphate (pH 7.6) with an ionic strength of 100 mM (adjusted by addition of potassium chloride) were lyophilized and dissolved in ^2^H_2_O (99.9 atom%, Spectra Stable Isotopes). NMR spectra obtained before and after lyophilization were identical, demonstrating that the proteins were not affected by this procedure. The protein concentration was determined by UV-visible spectroscopy using ε_410nm_ of 125,000 M^-1^cm^-1^ per heme for the oxidized state of the protein (Massey, 1959; Hartshorne et al., 2007). Samples containing 50 or 100 μM of STC and FccA were titrated against increasing concentrations of DmsE, MtrD, OTR, and ccNiR. The competition titration was performed with a sample of MtrA (50 μM) incubated with sufficient amount of FccA to have at least 90% of MtrA bound to FccA. Subsequently, increasing amounts of STC were added to the NMR tube, in order to detect any perturbation in STC signals. ^1^H-1D-NMR spectra were recorded after each addition. The chemical shifts of the signals corresponding to the methyl substituents of the hemes of STC and FccA were measured in each spectrum. These signals have been previously assigned to specific hemes in the structure, allowing the identification of the docking sites with its redox partners (Fonseca et al., 2009; Pessanha et al., 2009).

Nuclear magnetic resonance experiments were performed at 25°C on a Bruker Avance II spectrometer operating at 500 MHz equipped with a TXI probe. The proton spectra were calibrated using the water signal as an internal reference (Banci et al., 1995).

### Data Analysis and Binding Affinities

Chemical shift perturbations equal to or larger than 0.025 ppm were considered significant (Diaz-Moreno et al., 2005b). The chemical shift perturbations (Δδ_bind_) of the NMR signals from a cytochrome resulting from the complex formation with another cytochrome were plotted against the molar ratio (R) of [CytB]/[CytA]. The data were fitted using least squares minimization to a 1:1 binding model using equations (1) and (2) (Worrall et al., 2003):

(1) $$\Delta {\text{δ}}_{bind}=\frac{1}{2}\Delta {\text{δ}}_{bind}^{\infty }(A-\sqrt{\left(A^{2}-4R\right)})$$

(2) $$A=1+R+\frac{K_{d}({\left[Cyt_{A}\right]}_{0}R+{\left[Cyt_{B}\right]}_{0})}{{\left[Cyt_{A}\right]}_{0}{\left[Cyt_{B}\right]}_{0}}$$

where Δδ_bind_^∞^is the maximal chemical shift perturbation of the NMR signals resulting from the complex formation between Cyt_A_ and Cyt_B_, K_d_ is the dissociation constant, [Cyt_A_]_0_ is the initial concentration of Cyt_A_ and [Cyt_B_]_0_ is the stock concentration of Cyt_B_. When several methyl signals belonging to an individual heme were visible, the data obtained for all methyls were used to define the dissociation constant. Experimental uncertainty was estimated from the spectral resolution of the NMR data acquired.

### Spectroscopic Assay of Interprotein Electron Transfer

Electron transfer involving FccA from SOMR-1 was measured spectrophotometrically inside an anaerobic chamber using an UV–visible spectrophotometer (Shimadzu model UV-1800) to collect spectra in the range of 300–800 nm as previously described. Briefly, an approximate final concentration of 1 μM of each target protein was prepared in a 1 ml cuvette. Dilutions were made from stock solutions of DmsE, MtrD, OTR, ccNiR, and FccA in degassed 20 mM potassium phosphate buffer (pH 7.6) with 100 mM KCl. Each protein was reduced by addition of small amounts of a concentrated solution of sodium dithionite. The absorbance was monitored at 314 nm to avoid excess of reducing agent. Fumarate was added to the reduced protein solutions to a final concentration of ∼1 mM. Only when no change was observed in absorbance at 552 nm, the reaction would be initiated by the addition of 1 nM FccA. The spectral changes were monitored over time. Experiments were performed with constant stirring and the temperature was kept at 25°C using an external thermostatic bath.

### Protein Electrostatic Surface Potential Calculations

The structures of ccNiR (PDB code 3UBR; Youngblut et al., 2012) and the OTR (PDB code 1SP3; Mowat et al., 2004), were used to calculate the electrostatic potential at the surface of both proteins. Both proteins were set in their fully oxidized states, which were the experimental conditions used to study their interactions. The GROMOS 43A1 force field (Scott et al., 1999) was used to set the partial charges of the proteins and co-factors. The MEAD package (Bashford and Karplus, 1990), which solves the Poisson–Boltzmann equation for a system, was used to calculate the electrostatic potentials. The ionic strength used was 0 mM and the internal and external dielectric constants were set at 2 and 80, respectively. The electrostatic potential was mapped at the surface of the proteins using PyMOL (DeLano, 2003).

## Results

### NMR Titrations and Binding Affinities

**FIGURE 1:**
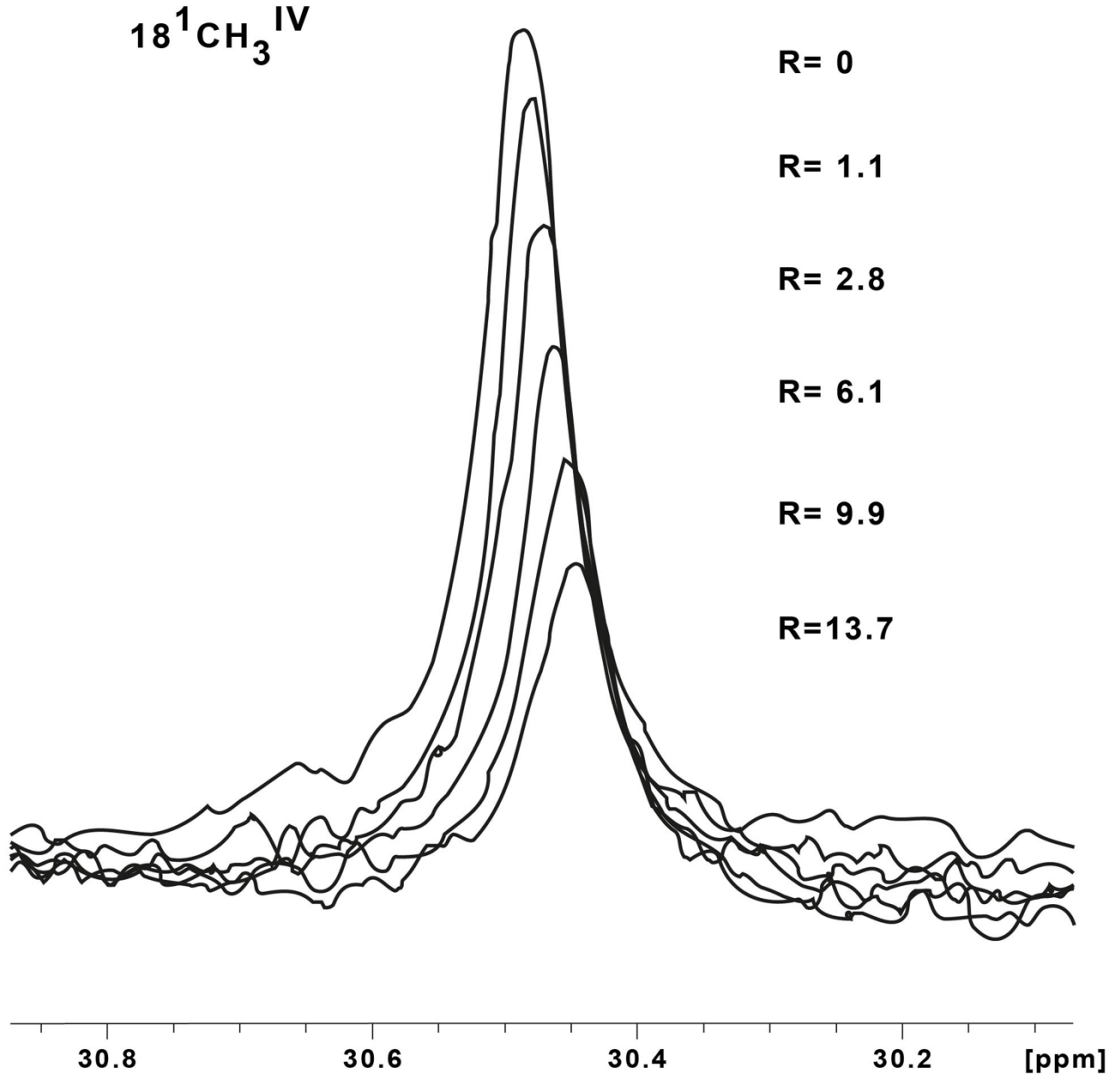
**^1^H-1D NMR spectral changes of the signal from methyl 18^1^ belonging to heme IV of small tetraheme cytochrome (STC) in the presence of increasing amounts of octaheme tetrathionate reductase (OTR), illustrating the data used in the chemical shift perturbation analysis.** The samples were prepared in 20 mM phosphate buffer pH 7.6, with 100 mM KCl, at 25°C. The methyl group is labeled using the IUPAC-IUB nomenclature for hemes. The Roman numeral corresponds to the order of heme binding to the polypeptide chain. The *R* values correspond to the molar ratios of [OTR]/[STC].

For electron transfer to occur at physiologically relevant rates between two cytochromes, the heme groups of donor and acceptor must be in close proximity (Zhang et al., 2008; Gray and Winkler, 2010). Therefore, when multiheme cytochromes bind in a configuration that is relevant for interprotein electron transfer, NMR spectroscopy can be used to detect this binding through observation of changes in the chemical shifts of signals belonging to the hemes near to the binding sites (Fonseca et al., 2013). This technique is thus highly suited to study interactions between the redox proteins found in the periplasmic space of SOMR-1, revealing the detailed organization of its trans-periplasmic redox network. **Figure 1** illustrates spectral changes for the 18^1^ methyl signal (IUPAC-IUB nomenclature) from heme IV (18^1^CH_3_^IV^) of STC in the presence of increasing amounts of OTR.

**FIGURE 2:**
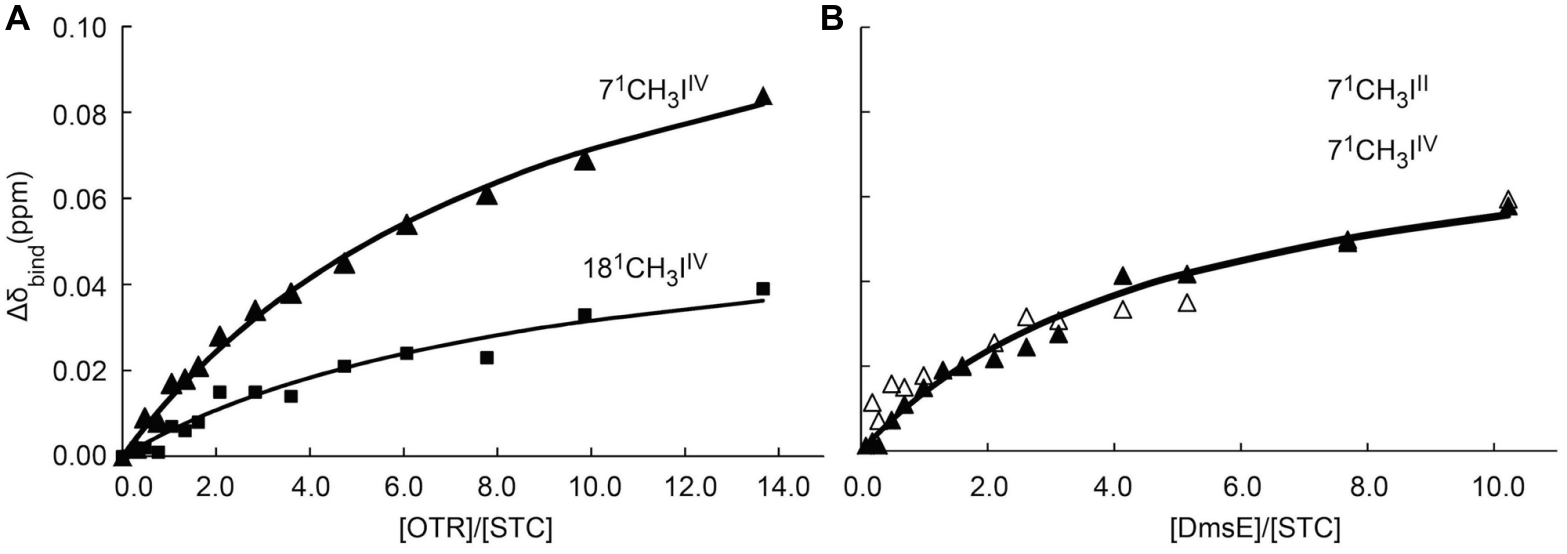
**Binding curves of periplasmic cytochromes from SOMR-1 that show interactions monitored by ^1^H-1D-NMR spectra: OTR and STC **(A)** and DmsE and STC **(B)**.** The chemical shift perturbations of the heme methyl signals are plotted as a function of the molar ratio of the interacting proteins. Solid triangles and solid squares represent the 7^1^ methyl and 18^1^ methyl of heme IV of STC, respectively; open triangles represent the 7^1^ methyl of heme II of STC. The solid lines represent the best global fit to the 1:1 binding model [equation (1)].

Chemical shift perturbations of the STC and FccA signals, resulting from binding to putative redox partners, were plotted against the molar ratio of redox partner:STC and redox partner:FccA (**Figure 2**). All the periplasmic pairs of proteins tested and K_d_ values of proteins that showed interactions are reported in **Table 2.** In some cases, such as the signals of the 2^1^ methyl of heme III or the 18^1^ methyl of hemes II and IV of STC during interaction with DmsE, the chemical shift perturbation of their signals is smaller than 0.025 ppm. These are therefore of insufficient magnitude for a confident estimation of binding parameters as indicated in the Section “Materials and Methods” and were not used in the calculations. The K_d_ values calculated are typical of weakly transient interactions as reported for other cytochromes (Diaz-Moreno et al., 2005a; Perkins et al., 2010; Bashir et al., 2011; Meschi et al., 2011; Fonseca et al., 2013).

**Table 2:** Pairwise interactions tested for cytochromes found in the periplasm of SOMR-1.

Cytochrome Complex	K_d_ (μM)	Docking Site
FccA and MtrA	35 (14)	Heme II
FccA and DmsE	–	
FccA and MtrD	–	
FccA and OTR	–	
FccA and ccNiR	–	
STC and MtrA	572 (5)	Heme IV
STC and DmsE	783 (227)	Hemes II,III, and IV
STC and MtrD	–	
STC and OTR	1600 (400)	Heme IV
STC and ccNiR	–	


*The “–” symbol indicates no interaction detected. Values in parenthesis are standard errors obtained from the diagonal of the covariance matrix arising from the fitting of the binding model to the experimental data*.

**FIGURE 3:**
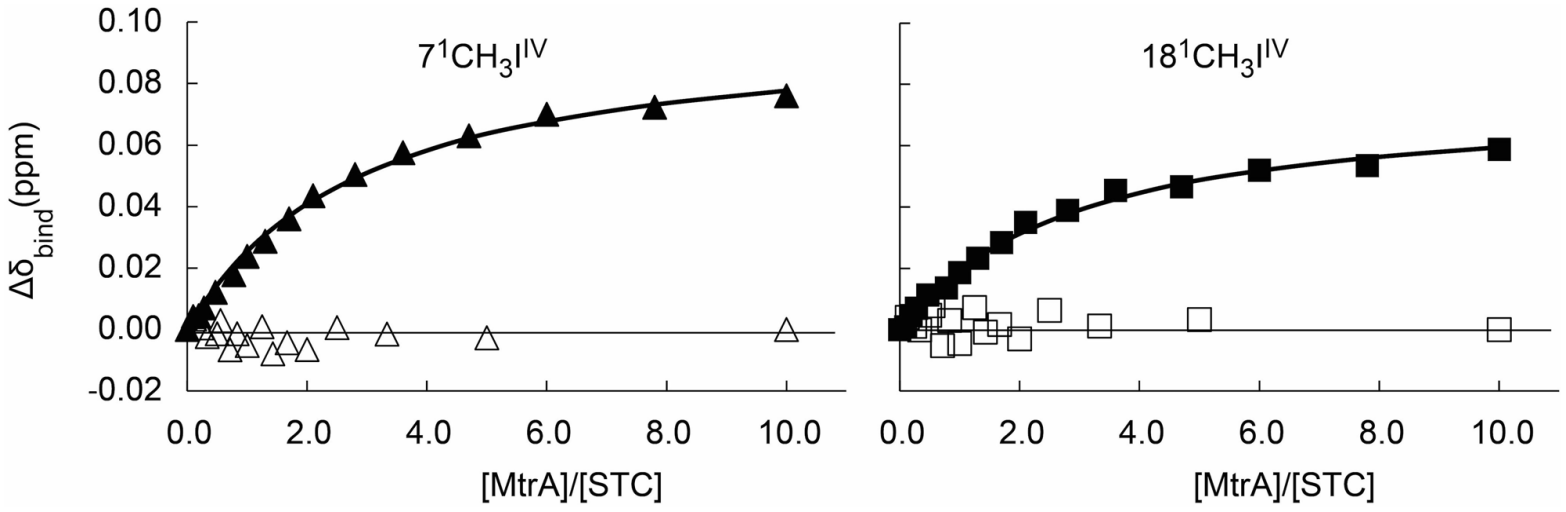
**Competition binding curves between STC and FccA with MtrA monitored by ^1^H-1D NMR spectra.** The chemical shift perturbations of the heme methyl signals are plotted as a function of the molar ratio of the interacting proteins. Filled symbols represent the experiment performed in the absence of FccA (Fonseca et al., 2013), whereas open symbols represent the experiment in the presence of 360 μM of FccA to ensure that more than 90% of MtrA is bound to FccA. The solid lines represent the best global fit to the 1:1 binding model [equation (1)] as reported by Fonseca et al. (2013).

Previous studies showed that both STC and FccA interact with the decaheme cytochrome MtrA and that the affinity between FccA and MtrA is much stronger than the affinity between STC and MtrA (Fonseca et al., 2013). Given that the three-dimensional structure of MtrA is not yet available, a competition binding assay monitored by ^1^H-1D-NMR was performed to study the docking place of STC and FccA with MtrA. In conditions where more than 90% of MtrA is bound to FccA, the chemical shift of the STC signals are not perturbed when the molar ratio of MtrA:STC is changed (**Figure 3**). This result shows that the presence of FccA bound to MtrA prevents the interaction between MtrA and STC, suggesting that the binding of STC and FccA at the surface of MtrA occurs in the same place or in close proximity.

The inverse competition binding assay with MtrA saturated with bound STC in the presence of increasing amounts of FccA added to the sample is not experimentally feasible. To reach more than 90% saturation of a sample with 50 μM of MtrA with STC would require concentrations of STC above 5 mM. Likewise, a similar experiment exploring the interactions of STC and FccA with CymA, another key protein for which a structure has not been reported in the literature, is not also experimentally feasible. The large dissociation constants reported for the interaction between STC and CymA or FccA and CymA (Fonseca et al., 2013) mean that achieving more than 90% saturation of 50 μM CymA with any of the partners would require concentrations of STC and FccA of 2.2 and 3.5 mM, respectively.

### Spectroscopic Assay of Interprotein Electron Transfer

**FIGURE 4:**
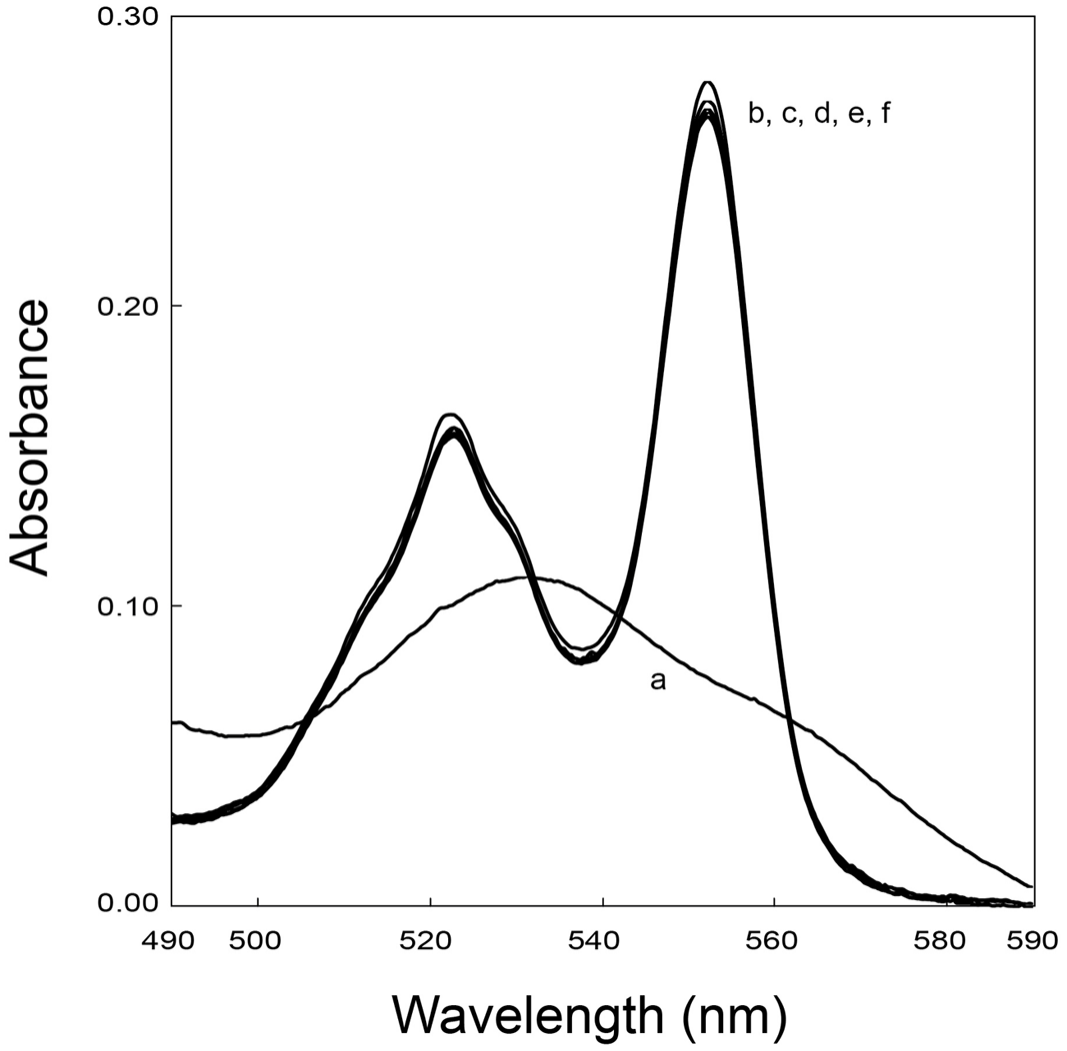
**UV–visible spectroscopy of reduced periplasmic cytochromes in the presence of excess fumarate and catalytic amounts of FccA illustrating the absence of electron transfer between OTR with FccA.** UV-visible spectrum of the cytochrome as purified (a), after reduction with sodium dithionite (b) and subsequent addition of fumarate (c). After addition of FccA to the mixture, spectra were acquired at 0 min (d), 2.5 min (e), and 5 min (f). The samples were prepared in 20 mM phosphate buffer pH 7.6, with 100 mM KCl, at 25°C.

UV-visible experiments were performed to confirm the interaction data obtained from NMR experiments involving FccA. The fumarate reductase activity of FccA was used to measure the re-oxidation of possible partner cytochromes. These experiments showed that FccA cannot re-oxidize MtrD, DmsE, OTR, and ccNiR (**Figure 4**), which is in agreement with the data obtained from NMR titrations.

### Electrostatic Calculations

**FIGURE 5:**
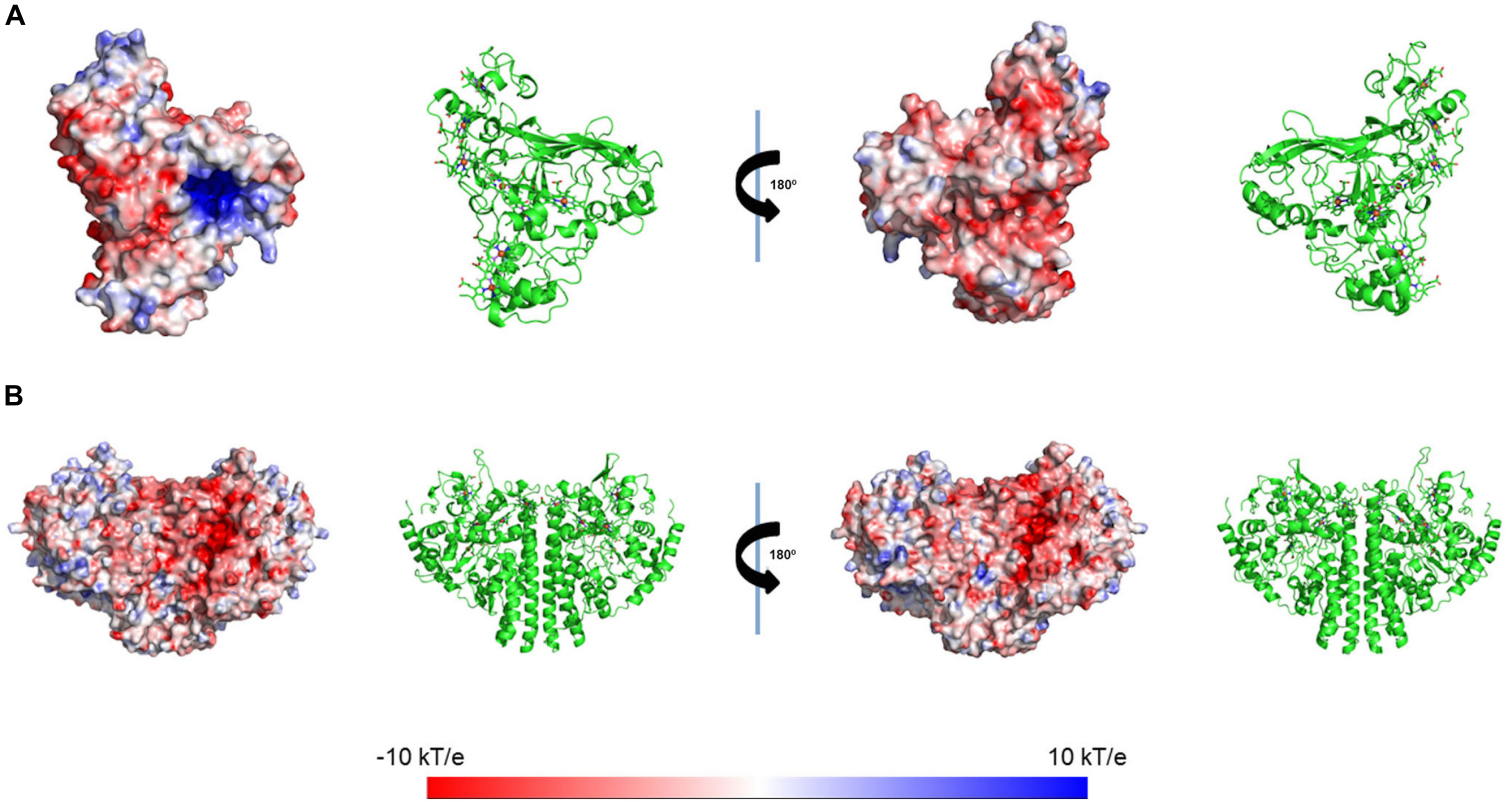
**Electrostatic potential mapping on the protein’s surface of **(A)** OTR (PDB code 1S3P) and **(B)** ccNiR (PDB code3UBR) from SOMR-1.** Electrostatic potentials were calculated considering a fully oxidized state for these cytochromes. The Roman numerals correspond to the order of heme binding motifs in the polypeptide chain.

The electrostatic potential at the surfaces of the enzymes ccNiR and OTR were calculated using the same procedure as previously used for STC and FccA (Fonseca et al., 2013). OTR presents an overall negative surface, with the exception of two regions, one near heme II (the catalytic heme) and the other one near heme VIII. In the case of ccNiR, the surface potential does not show a clear cut trend as in the case of OTR, with the exception of the region near heme I, which is the catalytic heme. The region around this heme is strongly negative (**Figure 5**).

## Discussion

The electron transfer pathways of the SOMR-1 to reduce Fe(III), DMSO, fumarate and nitrite are established by a variety of multiheme *c*-type cytochromes located at the inner membrane, periplasm and outer membrane. Biochemical studies showed that all of these routes have the common feature of being initiated by the oxidation of the quinone pool at the inner-membrane by the tetraheme *c*-type cytochrome, CymA (Schwalb et al., 2003; Marritt et al., 2012). The detailed understanding of the organization of the trans-periplasmic redox network has been compounded by two factors: spectroscopic signatures of *c*-type cytochromes are often overlapping or identical (Firer-Sherwood et al., 2011), and they form low-affinity complexes with fast dissociation rates (Crowley and Ubbink, 2003; Rudolph, 2007; Abresch et al., 2009). Nevertheless, recent studies have demonstrated that NMR spectroscopy is an effective technique to identify transient interactions between redox partners by monitoring the perturbation of the chemical shifts of heme signals (Fonseca et al., 2013). This method does not disturb the protein conformation because it is a soluble assay. Furthermore, it has the unique advantage of allowing the determination of docking regions between redox partners when resonance assignments of the interacting proteins are available.

A previous study revealed that STC and FccA can independently mediate electron transfer between CymA and MtrA leading to extracellular electron transfer (Fonseca et al., 2013). Since the three-dimensional structure of MtrA was only characterized at low resolution using SAXS (Firer-Sherwood et al., 2011), the docking with its redox partners STC and FccA cannot be modeled at this point. Notwithstanding, in this work the interaction of STC and FccA with MtrA was further characterized by a competition binding assay between STC and FccA. It revealed that these two proteins bind MtrA in the same or at least closely related locations, given that saturation of MtrA with FccA prevents the binding of STC.

This study also reveals that STC interacts transiently with DmsE and that the signals of hemes II, III, and IV are perturbed. Clearly, the interaction between STC and DmsE is different from that between STC and MtrA, which affects only signals of the heme IV of STC. Given the bracket shape of the structure of STC, if one considers the hemes ordered sequentially from top to bottom it can be envisaged that interaction with DmsE occurs via the lower external face of the bracket (Supplementary Figure S1).

The genome of SOMR-1 contains three homologues of the MtrCAB–OmcA complex, the MtrDEF complex, the DmsEFABGH complex and the complex coded by the genes SO_4357-62. Studies involving MtrA knockout strains showed that two of these complexes constitute alternative routes of electron flow to Fe(III) respiration (Coursolle and Gralnick, 2010). MtrA can be functionally replaced in ferric citrate reduction in the order MtrD > DmsE (Coursolle and Gralnick, 2012). Despite the high homology of SO4360 with MtrA, this decaheme cytochrome cannot functionally replace MtrA and requires its own porin SO4359 to function in metal reduction (Schicklberger et al., 2013). This hierarchy in the capacity for functional replacement of MtrA matches the sequence homology among these decaheme cytochromes (**Table 3**) but does not match the observed interactions with the major trans-periplasmic redox shuttles. MtrA interacts with STC and FccA that compete for the same binding site on the surface of MtrA. DmsE interacts with STC but not FccA, and MtrD does not interact with STC or FccA. Altogether, these data give strong indications that the dominant factor in the capacity of other periplasmic decaheme cytochromes to functionally replace MtrA is the matching with the MtrB porin to establish contact with the outer-membrane MtrC cytochrome, since neither STC nor FccA interact with MtrD.

**Table 3:** Sequence identity matrix for the periplasmic decaheme cytochromes from SOMR-1.

	MtrA	MtrD	DmsE	SO4360
MtrA	100	72	69	54
MtrD	++	100	60	49
DmsE	+		100	51
SO4360	-			100


*Sequences of the four decaheme cytochromes were retrieved from Pubmed-NCBI database and aligned using the Clustal 2.1 Multiple Sequence Alignment website. Values are presented in percentage. Below the diagonal is an indication of the relative ability to functionally replace MtrA according to Coursolle and Gralnick (2010)*.

Studies recently published by Sturm et al. (2015) showed that single mutants of STC and FccA have only minor phenotypic changes in their ability to reduce DMSO. However, the double mutant strain is unable to grow using this electron acceptor (Sturm et al., 2015). Those results suggest that both STC and FccA have a key role in the respiration of DMSO in SOMR-1 and the data reported in this work indicates that FccA does not interact directly with DmsE.

In this study, interactions involving two major terminal reductases involved in pathways for dissimilatory nitrate ammonification of SOMR-1, ccNiR, and OTR, were also explored (Berks et al., 1995; Simon, 2002; Einsle and Kroneck, 2004). While the pentaheme ccNiR catalyzes the reduction of nitrite (NO_2_^-^) to ammonium (NH_4_^+^), the octaheme OTR can reduce nitrite (NO_2_^-^) and hydroxylamine (NH_2_OH), as well as the sulfur compound tetrathionate (S_4_O_6_^2-^) (Atkinson et al., 2007). The results showed that heme IV of STC is perturbed upon interaction with OTR. The region around heme IV of STC displays the strongest negative surface, making it a good candidate to interact with OTR that displays positively charged potentials in various regions of its surface (**Figure 5**). By contrast, no interaction was observed between ccNiR and STC or FccA. The surface of ccNiR is predominantly weakly negative with a strongly negative region near the catalytic center, therefore discouraging interactions with the negatively charged STC or heme domain of FccA. This result is also consistent with a previous study showing that both wild-type SOMR-1 and the Δ*stc*Δ*fccA* mutant strain reduce nitrite at similar rates (Sturm et al., 2015).

Interestingly, all protein–protein interactions reported between STC and its putative physiological partners involve heme IV (Fonseca et al., 2013). Given that to perform its function of shuttling electrons between redox partners STC needs to charge and discharge, clearly this protein does not operate as a molecular wire. It functions more like an electronic cul-de-sac that forces electrons to enter and leave the cytochrome by the same heme. This enables *Shewanella* to transfer electrons in a controlled and efficient manner (maximum of four electrons can be transferred by STC) to specific proteins in the periplasmic space, rather than transfer randomly to any protein that may interact with STC. This minimizes the risk of diverting electrons to side redox pathways and production of radical species.

## Conclusion

**FIGURE 6:**
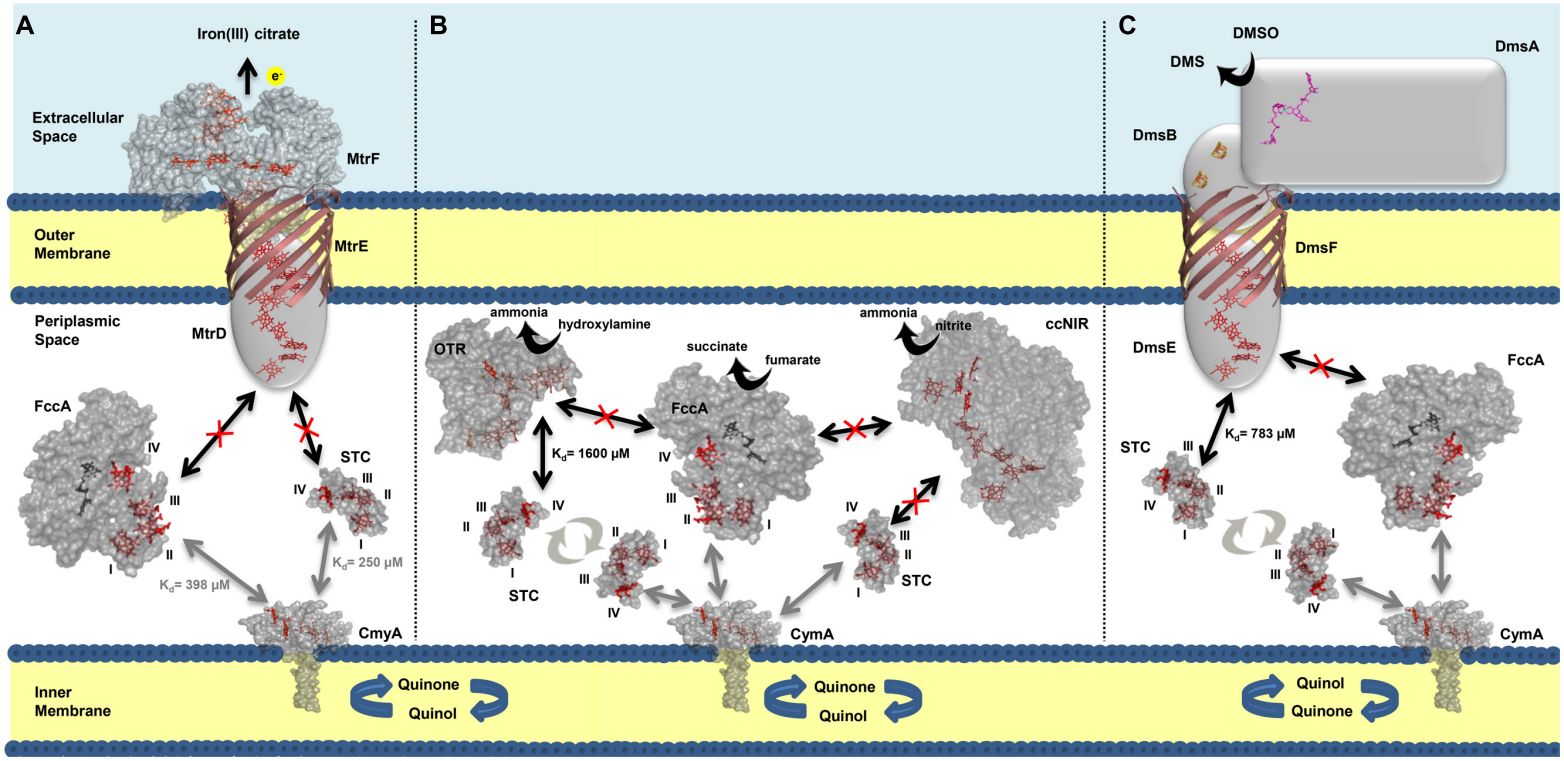
**Interactions between the most abundant periplasmic cytochromes (STC and FccA), and outer membrane complex MtrDEF **(A)**, periplasmic nitrite reductases OTR and ccNiR **(B)** and outer membrane complex DmsEFABGH **(C)**. The arrows in bold indicate the interactions that occur between the cytochromes and point to the possible docking site. Black arrows indicate data from this work and gray arrows indicate data previously reported (Fonseca et al., 2013). The dissociation constants (Kd) corresponding to each interaction are indicated next to their respective arrow. Interactions that were not detected experimentally are represented by a crossed-out arrow. The Roman numerals correspond to the order of heme attachment to the polypeptide chain. Cytochrome representations were made with PyMOL using the structures of STC (PDB code 1M1Q), FccA (PDB code 1D4D), OTR (PDB code 1S3P), and ccNiR (PDB code 3UBR). For CymA the model was made with SWISS-MODEL (Arnold et al., 2006; Kiefer et al., 2009) using as template the structure of NrfH (PDB code 2J7A)**.

We have elucidated the organization of the multi-branched periplasmic respiratory network of SOMR-1. NMR studies revealed that STC not only contributes to extracellular respiration of metals via interaction with MtrCAB–OmcA, but also to the reduction of nitrogen compounds and DMSO by interacting with OTR and DmsE, respectively (**Figure 6**). These results demonstrate that STC is a promiscuous periplasmic electron shuttle with a variety of redox partners. Notwithstanding, STC is clearly selective in the mode of interaction with its multiple partners that always appears to involve the participation of heme IV. Interestingly, in contrast to STC, no interaction was observed between FccA with MtrD, DmsE, OTR, and ccNiR. These results contrast with the functional redundancy that FccA provides for STC revealed by delayed or no growth with ferric citrate, DMSO or nitrite as electron acceptors (Sturm et al., 2015) in double deletion mutant studies. Further studies will reveal whether the differences arise from different metabolic regulation in the mutant strains or if FccA interacts indirectly with partners in those metabolic pathways.

## Conflict of Interest Statement

The authors declare that the research was conducted in the absence of any commercial or financial relationships that could be construed as a potential conflict of interest.

## Abbreviations

SOMR-1: Shewanella oneidensis MR-1
MFC: microbial fuel cell
STC: small tetraheme cytochrome
FccA: flavocytochrome c
OTR: octaheme tetrathionate reductase
ccNiR: Cytochrome c nitrite reductase.
