# Supplementary material for: Characterization of the periplasmic redox network that sustains the versatile anaerobic metabolism of Shewanella oneidensis MR-1
Source: Front Microbiol. 2015 Jun 29;6:665. doi: 10.3389/fmicb.2015.00665 (PMC4484225; doi:10.3389/fmicb.2015.00665)
Supplement: Supplementary file 1 [file Image_1.PDF]

Supplementary Material

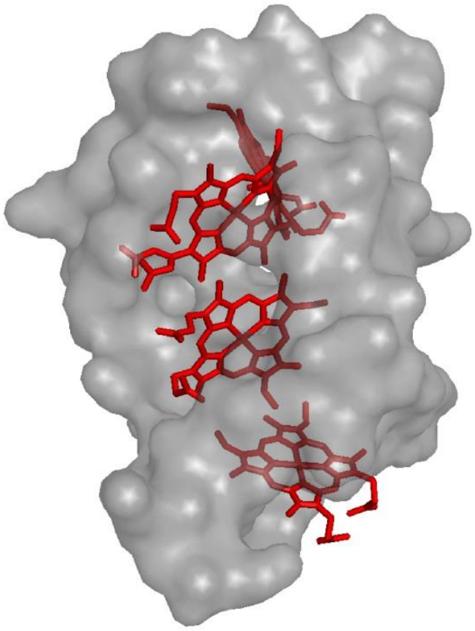

Figure S1 – STC represented with hemes ordered sequentially from top to bottom showing hemes II, III and IV exposed at the surface. The image was prepared with PyMOL using PDB code 1M1Q.
